# First evidence of asexual recruitment of *Pocillopora acuta* in Okinawa Island using genotypic identification

**DOI:** 10.7717/peerj.5915

**Published:** 2018-11-12

**Authors:** Yuichi Nakajima, Po-Shun Chuang, Nobuo Ueda, Satoshi Mitarai

**Affiliations:** 1 Marine Biophysics Unit, Okinawa Institute of Science and Technology Graduate University, Onna, Okinawa, Japan; 2 Okinawa Marine Science Support Section, OIST Marine Science Station, Onna, Okinawa, Japan

**Keywords:** Asexual reproduction, Coral, Genetic markers, Outdoor tank, Scleractinia

## Abstract

Okinawa Island is located near the center of the Nansei Islands (∼24–31°N), at a relatively high latitude for coral reefs. Nevertheless, more than 80 coral genera (over 400 species) are abundant in the Nansei Islands. Since March, 2017, scleractinian corals have been held in an outdoor tank at the OIST Marine Science Station at Seragaki, Onna with natural sea water flow-through in order to be used in molecular biological and physiological studies. In January, 2018, we found small pocilloporid-like colonies suspected to have originated asexually. We collected 25 small colonies and measured their sizes and weights. Also, we validated the classification and clonality of the colonies using a mitochondrial locus and nine microsatellite loci. Almost all of the small colonies collected in the outdoor tank were ≤1 cm in both width and height. The weight of dried skeletons ranged from 0.0287 to 0.1807 g. Genetic analysis determined that they were, in fact, *Pocillopora acuta*. Only one mitochondrial haplotype was shared and two microsatellite multilocus genotypes were detected (20 colonies of one and four colonies of the other). The mitochondrial haplotype and one microsatellite multilocus genotype for 20 colonies corresponded to those of one *P. acuta* colony being kept in the tank. One small colony matched both multilocus genotypes. This may have been a chimeric colony resulting from allogenic fusion. These small colonies were not produced sexually, because the only potential parent in the tank was the aforementioned *P. acuta* colony. Instead, they were more likely derived from asexual planula release or polyp bail-out. Corals as *Pocillopora acuta* have the capacity to produce clonal offspring rapidly and to adapt readily to local environments. This is the first report of asexual reproduction by planulae or expelled polyps in *P. acuta* at Okinawa Island.

## Introduction

**Figure 1:**
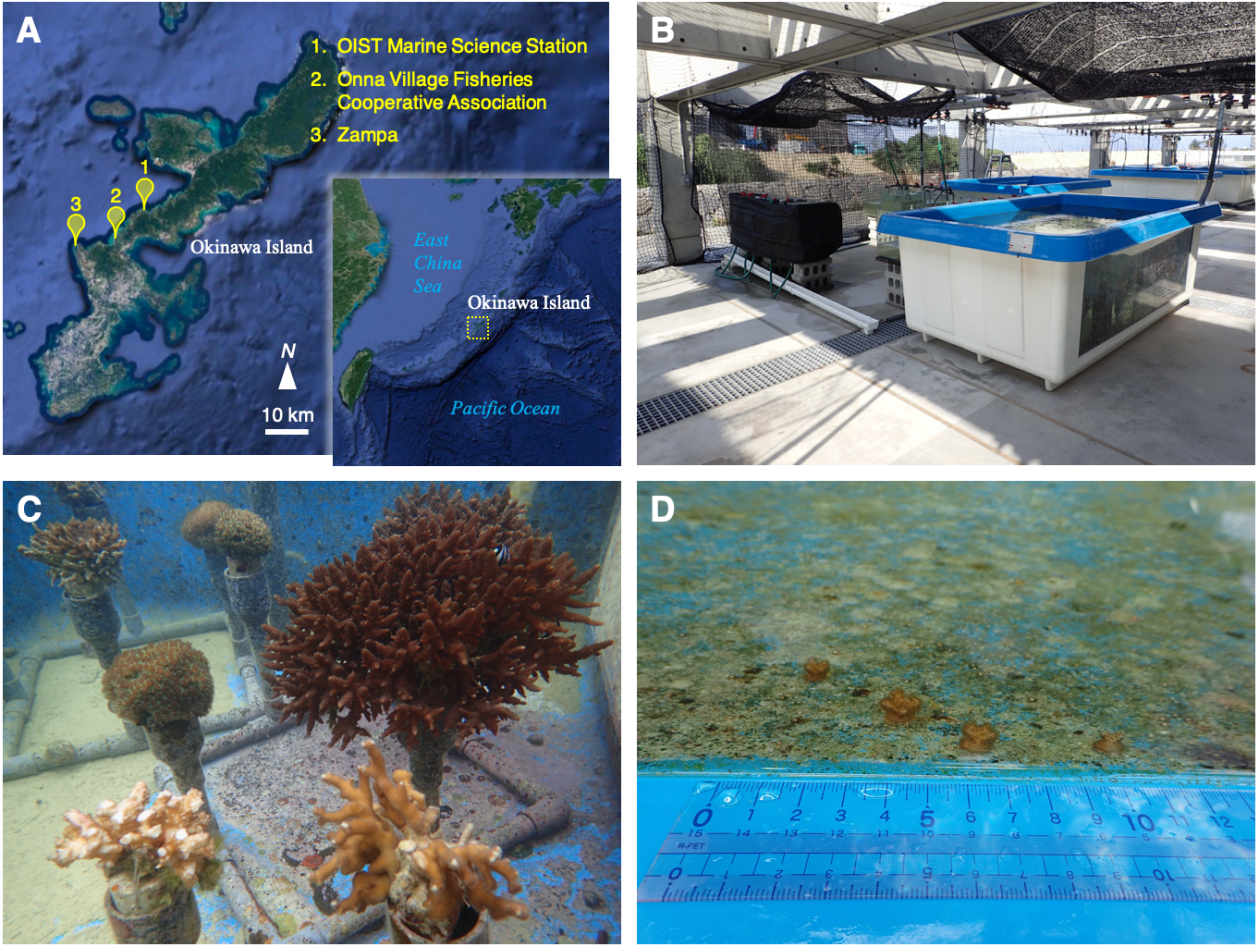
Location, outdoor tank, and coral colonies. (A) A map of the Nansei Islands and the location of the OIST Marine Science Station, Onna Village Fisheries Cooperative Association, and Zampa in Okinawa Island. Original maps were downloaded from Google Earth. Map data: Google Earth, Image Landsat/Copernicus, Data SIO, NOAA, US Navy, NGA, GEBCO. (B, C) Coral colonies maintained under natural sea water flow in the outdoor tank. (D) Small colonies were discovered in the tank on January, 2018. These photographs in B, C, and D were taken by Yuichi Nakajima.

The Nansei Islands, spanning approximately 1,000 km, are located to the north of the coral triangle in the East China Sea and the Pacific Ocean (Fig. 1A). This is relatively high latitude (∼24–31°N) for coral reefs, and includes the northern reef limit in the northern hemisphere (Yamano, 2008). Over 80 coral genera (over 400 species) are abundant in the area (Nishihira & Veron, 1995; The Japanese Coral Reef Society & Ministry of the Environment, 2004). The geographic reef range and coral species diversity appear to be enhanced by the Kuroshio Current and its branch currents that flow from the east side of the Philippines (Nishihira & Veron, 1995). However, like many tropical reefs, coral populations in the Nansei Islands are suffering from anthropogenic disturbances. Since the mass coral bleaching event in 1998, coral bleaching has often been observed in the region (Loya et al., 2001; Hongo & Yamano, 2013; Harii et al., 2014). Erosion of red soil from sugar cane farming has destroyed water quality in coastal waters (Hasegawa et al., 1999), and outbreaks of coral-eating, crown-of-thorns starfish, *Acanthaster planci* (*Acanthaster* cf. *solaris*) have caused further damage to coral reefs (Yamaguchi, 1986; Nakamura et al., 2015). Multiple environmental stresses at global and local scales are expected to degrade coral reefs in the region for the foreseeable future (Harii et al., 2014).

Population retention and larval recruitment of corals have been studied in the Nansei Islands (Loya et al., 2001; Nakamura & Sakai, 2010; Van Woesik et al., 2011). Monitoring of coral coverage and coral composition over long periods is fundamental to understand the circumstances of living corals and to support future reef conservation efforts. In addition to monitoring numbers and densities of coral colonies, it is also important to assess larval coral recruitment. However, the recruitment process is complex in corals because of multiple reproductive strategies involving both sexual and asexual reproduction (Harrison & Wallace, 1990). The annual spawning season varies among genera/species, even in the same region (Shlesinger & Loya, 1985; Okubo et al., 2013). Among sexually reproducing species, both hermaphroditic taxa, e.g., *Acropora*, and dioecious species, e.g., *Galaxea fascicularis* are known (Babcock et al., 1986; Keshavmurthy et al., 2012). However, clonal replicates, produced by fragmentation and asexual planula, may also contribute to reef formation as well (Harrison & Wallace, 1990). Thus, reproductive strategy plays a key role in understanding population retention and maintenance.

Okinawa Island (∼26–27°N) comprises ∼1,200 km^2^ and is located near the center of the archipelago (Fig. 1A). It is currently home to over 1.3 million people and the population is concentrated in the south and middle sections of the island, which are relatively developed. The central and northwestern coastal area has also been designated as an “Okinawan semi-national coastal park” to conserve geological features and marine coastal fauna, and to limit development of the coast and to ensure the survival of marine species. In July, 2016, Okinawa Institute of Science and Technology Graduate University (OIST) inaugurated the OIST Marine Science Station at Seragaki, Onna, in the central west coast of Okinawa to advance marine research (Fig. 1A). This station possesses indoor and outdoor tanks with a constant flow of natural and sand-filtered sea water to hold various marine organisms. In March, 2017, we started to hold multiple coral species for molecular biological and physiological studies. Ten months after, we found small pocilloporid-like colonies in an outdoor tank. It is difficult to identify coral species morphologically and their plasticity has resulted in taxonomic confusion (Paz-García et al., 2015); hence, genetic markers have been used for classification (Pinzón et al., 2013; Schmidt-Roach et al., 2013). In this study, we validated the classification and clonality of these small colonies by comparing them with adult colonies in the tank using genetic methods, and here we present a coral population dynamics study in a miniaturized, artificial world.

## Materials and Methods

### Coral keeping and collection of small corals

We started to keep coral colonies of *Acropora*, *Galaxea*, *Pocillopora*, and *Stylophora* in an outdoor tank with constantly flowing natural sea water, in March, 2017 (Figs. 1B and 1C). These colonies were purchased from Onna Village Fisheries Cooperative (Fig. 1A). Also, we collected and kept morphological *Pocillopora verrucosa* colonies in Zampa (Fig. 1A), on the west coast of Okinawa Island commencing in May, 2017. The collection of corals was approved by Okinawa prefecture (permit number: 28-80). The list of corals that were kept in the tank is shown in File S1. Although we did not observe spawning of these corals, we spotted small pocilloporid-like corals adhering to the side wall of the tank (Fig. 1D). We confirmed over 40 small colonies on the tank wall in January, 2018. Using a flathead screwdriver, we collected 25 small corals, which were readily accessible on the inner wall of the tank. A tip of mature pocilloporid corals was also collected like as these small corals. However, morphological *P. verrucosa* colonies from Zampa died before the procedure started. After samples were soaked in a mixture of ATL buffer and Proteinase K in DNeasy Blood & Tissue Kit (Qiagen, Hilden, Germany) for extraction of genomic DNA (see below), we preserved the 25 coral skeletons in ethanol and then dried them for more than a week at room temperature. Then, we measured the maximum width and height of each skeleton using an electronic caliper E-PITA15 (Nakamura Mfg, Matsudo-shi, Japan) and their weight on an Analytical Balance ME54 (Mettler Toledo, Columbus, OH, USA). The sum of individual width and height was calculated for each colony. We calculated the correlation of size (sum of the width and height) with weight by regression analysis using statistical package R ver. 3.4.4 (R Core Team, 2016) and determined the microsatellite multilocus genotypes (see below).

### Genotyping of mitochondrial haplotypes

It is impossible to identify these coral species as recruits by eye, due to the complex relationship between morphology and genetic species (Schmidt-Roach et al., 2013; Schmidt-Roach et al., 2014a; Schmidt-Roach et al., 2014b; Nakajima et al., 2017). Genomic DNA from mature pocilloporid colonies and the 25 small colonies was extracted using a DNeasy Blood & Tissue Kit, following the standard protocol. We conducted genotyping using the mitochondrial open reading frame region (mtORF), which is useful for identification of genetic types of pocilloporids (Flot et al., 2008; Flot et al., 2011). Mitochondrial haplotypes were confirmed by sequencing the mtORF region. PCR reaction mixtures (10 µL) contained template DNA (<50 ng/µL), AmpliTaq Gold 360 Master Mix (Thermo Fisher Scientific), and primers (final concentration: 2 µM for each primer) for mtORF: FATP6.1 (5′-TTTGGGSATTCGTTTAGCAG-3′) and RORF (5′-SCCAATATGTTAAACASCATGTCA-3′). The PCR protocol consisted of 94 °C for 1 min, followed by 40 cycles at 94 °C for 30 s, 53 °C for 30 s, and 72 °C for 75 s, with a final extension at 72 °C for 5 min using thermal cycler, Thermal Cycler Dice Touch TP350 (Takara, Kusatsu, Japan). PCR products were cleaned with Exonuclease I (Takara) and Shrimp Alkaline Phosphatase (Takara). Sequencing was performed by Macrogen Japan, and sequences obtained were aligned with mtORF sequences reported in Nakajima et al. (2017) and Flot et al. (2011) to identify the genetic species of *Pocillopora* and *Stylophora*, respectively. We could not obtain the sequence of morphological *P. verrucosa* colonies collected from Zampa, because these colonies had died previously.

### Scoring of microsatellite multilocus genotypes

Nine microsatellite loci developed for *Pocillopora* by Nakajima et al. (2017) were used to investigate the relationship between potential parental colonies and the small coral colonies discovered on the tank wall. These nine loci have perfect repeats (see Table 1 in Nakajima et al., 2017). Information about microsatellite loci is presented in Table 1. For scoring of microsatellite genotypes of each colony, the PCR reaction mixture (5 µL) contained template DNA (<50 ng/µL), AmpliTaq Gold 360 Master Mix, and three primers for each locus: a non-tailed primer (0.5 µM), another primer with a sequence tail of U19 (5′-GGTTTTCCCAGTCACGACG-3′) (0.5 µM), and a U19 primer (0.5 µM) fluorescently labeled with FAM, VIC, or NED. The PCR protocol consisted of 95 °C for 9 min, followed by 35 cycles at 95 °C for 30 s, 56 °C for 30 s, and 72 °C for 1 min, with a final extension at 72 °C for 5 min using the thermal cycler. PCR products were analyzed using an ABI 3130xl capillary DNA sequencer (Thermo Fisher Scientific, Waltham, MA, USA) with GeneScan 600 LIZ size standards (Thermo Fisher Scientific) to identify genotypes by the length of the amplicon. Fragment size and intensity were confirmed using Geneious version 9.0.4 (Biomatters, Auckland, New Zealand). We calculated the probability of identity (*P*_ID_) value using multilocus genotypes obtained from potential parental colonies and small colonies using GenAlEx ( Peakall & Smouse, 2012) for the possibility of clonal occurrence.

**Table 1:** Characteristics of microsatellite loci used in this study. Locus name, forward and reverse primer sequences, microsatellite genotypes (fragment length), fluorescent dye for U19, and GenBank accession number for each locus in *Pocillopora acuta* (Type 5 in mtORF) (see Nakajima et al., 2017). One colony consistently displayed two genotypes in the fragment analysis.

**Locus**	**Forward primer sequence (5′−3′)**	**Reverse primer sequence (5′−3′)**	**Microsatellite genotypes**		**Fluorescent dye for U19**	**Accession No.**
			**20 colonies**	**4 colonies**		
Psp_02	CTGTGCTGGAATTCCCCTTA	U19-AGCCTACGGCGCAATAGTAG	261/281	234/234	FAM	LC222432
Psp_16	CCCGCTGCTGAGTAAGAATC	U19-AGAGAAACTGCAAAACCGC	181/181	187/188	FAM	LC222434
Psp_18	CACACGTTTTATGACAACGGA	U19-ATAAGCCGTAGGCCCTGTCT	307/327	275/303	FAM	LC222435
Psp_23	ACCATTGCCATCACTGTTCA	U19-TTCATTCATTCGTATTGGCG	158/158	157/165	VIC	LC222436
Psp_29	TTTCGTACCAAAATCCAGGC	U19-TTTTTCAGTCGCAAGAGGC	257/262	257/257	VIC	LC222437
Psp_32	AAGCACGCAATTCAGCCTAT	U19-AGCCTAAGACGAATCGAGCA	127/147	127/127	VIC	LC222438
Psp_33	CCATTTCCCGAATCTCTCTC	U19-CTCGTCGCCCAGATATAAA	258/266	256/270	NED	LC222439
Psp_39	TCTTTACAGCACAGGAGCCA	U19-TTTTTCTTGCGGTCCAATTC	121/121	121/121	NED	LC222441
Psp_48	TGTAAATTCAAGAGAATGGGCA	U19-GTTTCCTGATGGTGTTCT	183/183	183/191	NED	LC222443

## Results

**Figure 2:**
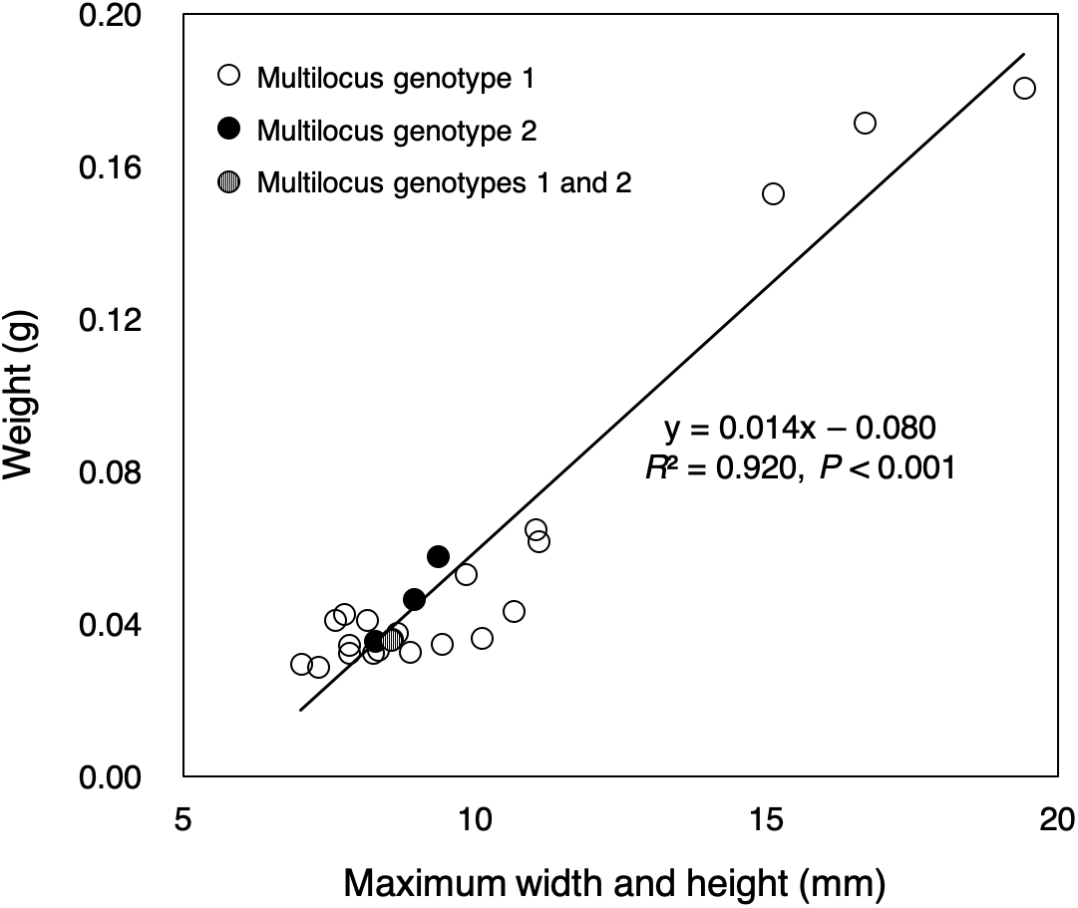
Sizes and weights of 25 colonies collected. The color of the circle corresponds to the microsatellite genotypes (white circle: multilocus genotype 1 for 20 colonies, black circle: multilocus genotype 2 for four colonies), but one colony had two multilocus genotypes (circle with white and black stripes).

Almost all of small colonies collected in the outdoor tank were ≤1 cm in width and height, ranging from 7.02 to 19.42 mm in size (sum of the width and height) and from 0.0287 to 0.1807 g in weight, respectively (Fig. 2, File S2). Size and weight were highly correlated (*P* < 0.001, *R*^2^ = 0.920). All small colonies were identified as *Pocillopora* corals according to a genetic analysis of mitochondrial haplotype. There were at least three genetic species of potential adult pocilloporid colonies in the tank: *Pocillopora* Type 1 (*Pocillopora meandrina*/*eydouxi* species complex), Type 3 (*P. verrucosa*), Type 5 (*Pocillopora acuta*, known as *Pocillopora damicornis* Type β in Schmidt-Roach et al. (2013), and *Stylophora* C (see File S1) following the nomenclature of Pinzón et al. (2013) for *Pocillopora* and Flot et al. (2011) for *Stylophora*. However, all mtORF sequences of the 25 small coral colonies were *Pocillopora* Type 5. Only one mtORF haplotype was shared between potential adult colony of *P. acuta* and the 25 small colonies (GenBank accession number LC390046), and two microsatellite multilocus genotypes were detected (20 colonies of one and four of the other) (Table 1). One small colony matched both multilocus genotypes. Peaks showing four or fewer microsatellite alleles were found per microsatellite locus (File S3). One microsatellite multilocus genotype for 20 colonies matched that of a potential adult colony. The *P*_ID_ value was 2.9e^−4^ in among the 25 colonies (one potential adult colony, 20 colonies with genotype 1 and four colonies with genotype 2; the colony with both multilocus genotypes was removed). Even in the calculation using only 21 colonies (one potential adult colony and 20 colonies with genotype 1), the *P*_ID_ value was 7.4e^−3^.

## Discussion

This study reports the first evidence for asexual recruitment in *Pocillopora* corals at Okinawa Island. These *Pocillopora* colonies probably were not all released synchronously, judging from the size differences among them, even though they shared the same multilocus genotype. The multilocus genotype for the 20 colonies matched that of a colony placed in the tank in March, 2017. From the *P*_ID_s of potential parental colonies and small colonies, we assume that coral recruits were derived from asexual reproduction. We tried to estimate the index of *P*_SEX_ using RClone (Bailleul, Stoeckel & Arnaud-Haond, 2016) to assess the possibility of clonality, but there were only two multilocus genotypes, resulting in such low statistical power that we could not calculate appropriate *P*_SEX_ values in the population (see also Arnaud-Haond et al., 2007; Nakajima et al., 2016). Meanwhile, another multilocus genotype (for four colonies) could not be assigned to any colony in the tank. One possible explanation is that this multilocus genotype may have been derived from a colony collected in Zampa and identified as *P. verrucosa* (see File S1); however, it died before it could be genotyped. This explanation is based on the assumption that the deceased colonies were actually *P. acuta*. However, there is a possibility that recruits came from outside the tank because of natural seawater without filtration. Interestingly, one small colony matched both multilocus genotypes. We suspected contamination and extracted genomic DNA again; however, the result was consistent. Thus, this may have been a chimeric colony resulting from allogeneic fusion of planula larvae from two different colonies that were not fused when branches of adult colonies were tried to be fused in *P. damicornis* (Hidaka, 1985; Hidaka et al., 1997).

Our data show that the small colonies found in the tank had an asexual origin and they were not produced sexually. This means that *P. acuta* may be able to spawn clonal offspring rapidly and to adapt to local environments easily. It has been confirmed that *P. acuta* releases sperm during sexual reproduction (e.g., Schmidt-Roach et al., 2014a; Schmidt-Roach et al., 2014b), but that they also produce clonemates (e.g., Torda et al., 2013; Gélin et al., 2017). Clonality of *P. acuta* was documented in our previous population genetic analysis for corals in the Nansei Islands. A population at Ueno, Miyako Island showed clonality with only 10 different multilocus lineages, among 29 colonies (Nakajima et al., 2017). Powerful waves during tropical storms may cause fragmentation of *P. verrucosa* colonies (Aranceta-Garza et al., 2012), but only if fragment branches can survive long enough to reattach on the sea bottom can they give rise to viable new colonies. We have observed that for fragile coral colonies of *G. fascicularis*, fragmented branches formed colonies of small, clonal-like branches with a original branch. Actually, clonemates of *G. fascicularis* had been often found in the Nansei Islands (Nakajima et al., 2015; Nakajima et al., 2016). However, in our outdoor tank, most colonies of *P. acuta* appear to result from asexual planula larvae. If these small colonies had been derived from fragmentation, fragmented branches would have sunk to the bottom, rather than attaching to the side wall, especially near the surface (see Fig. 1D). This result suggests that clonal colonies of *P. acuta* in Okinawa have been maintained asexually with planula larvae, though the frequency of fragmentation is not yet known. In Eastern Australia, *P. acuta* also releases brooded asexual larvae (Schmidt-Roach et al., 2013) as do morphological *P. damicornis* in Western Australia (Stoddart, 1983; Ayre & Miller, 2004) and Taiwan (Yeoh & Dai, 2010). Our research provides additional evidence that clonal reproduction is one of the reproductive strategies adopted by *P. acuta*.

However, polyp bail-out in *Pocillopora* must be considered in stressful local environments (P-S Chuang, Y Nakajima & S Mitarai, 2018, unpublished data). Coral polyps are expelled into the water column under extreme stress (*Oculina patagonica*: Kramarsky-Winter, Fine & Loya, 1997; *Seriatopora hystrix*: Sammarco, 1982; *P. damicornis*: Fordyce, Camp & Ainsworth, 2017). It is possible that expelled polyps have settlement competency like that of planula larvae; however, that possibility requires validation. In addition, physiological experiments are needed to estimate the timing and condition of planula release and polyp bail-out. With the data collected here, it is impossible to determine whether these small clonal colonies were from released planula larvae or from bail-out due to environmental conditions. Our observations help to better understand coral physiology and ecology of sustainable coral populations and they provide new data about the reproductive characteristics of corals inhabiting the Nansei Islands.

## Conclusion

In this study, we found evidence of asexual recruitment that is supported by genetic analysis. These *Pocillopora* colonies probably were not released synchronously judging from size differences among the colonies. This is the first report of asexual reproduction in a species of *Pocillopora* at Okinawa Island. One small colony appeared to be a chimeric colony resulting from the allogeneic fusion of planula larvae. This research improves our understanding of coral physiology and life history, and contributes to sustainability of coral populations in the Nansei Islands.

##  Supplemental Information

10.7717/peerj.5915/supp-1 File S1 A list of corals kept in the outdoor tank Mitochondrial DNA types of corals were defined following Nakajima et al. (2016) for *Galaxea fascicularis*, Pinzón et al. (2013) for genus *Pocillopora* and Flot et al. (2013) for *Stylophora pistillata*.

10.7717/peerj.5915/supp-2 File S2 Size and weight of each colony Size means sum of maximum width and height of each colony. Bold means maximum or minimum value in the column.

10.7717/peerj.5915/supp-3 File S3 Microsatellite peaks of colonies including the allogenically fused colony These peaks were output from Geneious after fragment analysis on an ABI 3130xl capillary DNA sequencer. A: Psp_02 (blue), Psp_23 (green), and Psp_33 (black). B: Psp_16 (blue), Psp_29 (green), and Psp_39 (black). C: Psp_18 (blue), Psp_32 (green), and Psp_48 (black). Specific information for each locus is shown in Table 1.

## Additional Information and Declarations

### Field Study Permissions

The following information was supplied relating to field study approvals (i.e., approving body and any reference numbers): The collection of corals was approved by Okinawa prefecture (permit number: 28-80).

### DNA Deposition

The following information was supplied regarding the deposition of DNA sequences: Mitochondrial ORF sequence of *Pocillopora acuta* described here is accessible via GenBank accession number LC390046.

### Data Availability

The following information was supplied regarding data availability: Colonial size and weight data are included in File S2. Microsatellite genotypes data is in Table 1.
