# Supplementary material for: First evidence of asexual recruitment of Pocillopora acuta in Okinawa Island using genotypic identification
Source: PeerJ. 2018 Nov 12;6:e5915. doi: 10.7717/peerj.5915 (PMC6237110; doi:10.7717/peerj.5915)
Supplement: File S1 — Mitochondrial DNA types of corals were defined following Nakajima et al. (2016) for Galaxea fascicularis, Pinzón et al. (2013) for genus Pocillopora and Flot et al. (2013) for Stylophora pistillata. [file peerj-06-5915-s001.docx]

| **Colony No.** | **Species** | **Mitochondrial DNA type** | **Retailer or sampling site** |
| --- | --- | --- | --- |
| 1 | *Acropora* cf. *samoensis* | Not analyzed | Onna Village Fisheries Cooperative Association |
| 2 | *Acropora* cf. *samoensis* | Not analyzed | Onna Village Fisheries Cooperative Association |
| 3 | *Acropora* sp. | Not analyzed | Onna Village Fisheries Cooperative Association |
| 4 | *Acropora* sp. | Not analyzed | Onna Village Fisheries Cooperative Association |
| 5 | *Galaxea fascicularis* | mt-S | Onna Village Fisheries Cooperative Association |
| 6 | *Galaxea fascicularis* | mt-S | Onna Village Fisheries Cooperative Association |
| 7 | *Galaxea fascicularis* | mt-S | Onna Village Fisheries Cooperative Association |
| 8 | *Galaxea fascicularis* | mt-S | Onna Village Fisheries Cooperative Association |
| 9 | *Pocillopora* cf. *meandrina* | Type 1 | Onna Village Fisheries Cooperative Association |
| 10 | *Pocillopora* *verrucosa* | Type 3 | Onna Village Fisheries Cooperative Association |
| 11 | *Pocillopora* *verrucosa* | Type 3 | Onna Village Fisheries Cooperative Association |
| 12 | *Pocillopora* *acuta* | Type 5 | Onna Village Fisheries Cooperative Association |
| 13 | *Pocillopora* cf. *verrucosa* | Not analyzed (died) | Zampa, Okinawa |
| 14 | *Pocillopora* cf. *verrucosa* | Not analyzed (died) | Zampa, Okinawa |
| 15 | *Pocillopora* cf. *verrucosa* | Not analyzed (died) | Zampa, Okinawa |
| 16 | *Pocillopora* cf. *verrucosa* | Not analyzed (died) | Zampa, Okinawa |
| 17 | *Stylophora pistillata* | C | Onna Village Fisheries Cooperative Association |
