# Supplementary material for: First evidence of asexual recruitment of Pocillopora acuta in Okinawa Island using genotypic identification
Source: PeerJ. 2018 Nov 12;6:e5915. doi: 10.7717/peerj.5915 (PMC6237110; doi:10.7717/peerj.5915)
Supplement: File S2 — Size means sum of maximum width and height of each colony. Bold means maximum or minimum value in the column. [file peerj-06-5915-s002.docx]

| **Colony** | **Genotype** | **Maximum width (mm)** | **Height (mm)** | **Size (mm)** | **Weight (g)** |
| --- | --- | --- | --- | --- | --- |
| 1 | 2 | 5.09 | 3.21 | 8.30 | 0.0354 |
| 2 | 1 | 5.48 | 2.86 | 8.34 | 0.0331 |
| 3 | 1 | 6.69 | 4.41 | 11.10 | 0.0616 |
| 4 | 1 | 5.91 | 4.21 | 10.12 | 0.0364 |
| 5 | 1 | 5.72 | 2.94 | 8.66 | 0.0375 |
| 6 | 1 | 8.22 | 6.90 | 15.12 | 0.1528 |
| 7 | 1 | 5.82 | 5.22 | 11.04 | 0.0647 |
| 8 | 2 | 7.29 | 2.08 | 9.37 | 0.0576 |
| 9 | 1 | 5.18 | 2.13 | 7.31 | **0.0287** |
| 10 | 1 | **10.76** | 5.93 | 16.69 | 0.1715 |
| 11 | 1 | 5.22 | 4.22 | 9.44 | 0.0346 |
| 12 | 1 | 6.03 | 4.63 | 10.66 | 0.0434 |
| 13 | 1 | 5.04 | 3.85 | 8.89 | 0.0327 |
| 14 | 2 | **4.35** | 4.22 | 8.57 | 0.0360 |
| 15 | 2 | 5.22 | 3.74 | 8.96 | 0.0463 |
| 16 | 1 | 6.04 | 3.81 | 9.85 | 0.0530 |
| 17 | 1 | 5.68 | 2.16 | 7.84 | 0.0344 |
| 18 | 1 and 2 | 4.90 | 3.67 | 8.57 | 0.0357 |
| 19 | 1 | 5.23 | 2.62 | 7.85 | 0.0323 |
| 20 | 1 | 6.28 | **1.87** | 8.15 | 0.0410 |
| 21 | 1 | 8.50 | **10.92** | **19.42** | **0.1807** |
| 22 | 1 | 4.61 | 3.64 | 8.25 | 0.0324 |
| 23 | 1 | 5.45 | 2.16 | 7.61 | 0.0409 |
| 24 | 1 | 5.55 | 2.20 | 7.75 | 0.0425 |
| 25 | 1 | 4.66 | 2.36 | **7.02** | 0.0296 |
