# Supplementary figures and images for: First evidence of asexual recruitment of Pocillopora acuta in Okinawa Island using genotypic identification
Source: PeerJ. 2018 Nov 12;6:e5915. doi: 10.7717/peerj.5915 (PMC6237110; doi:10.7717/peerj.5915)

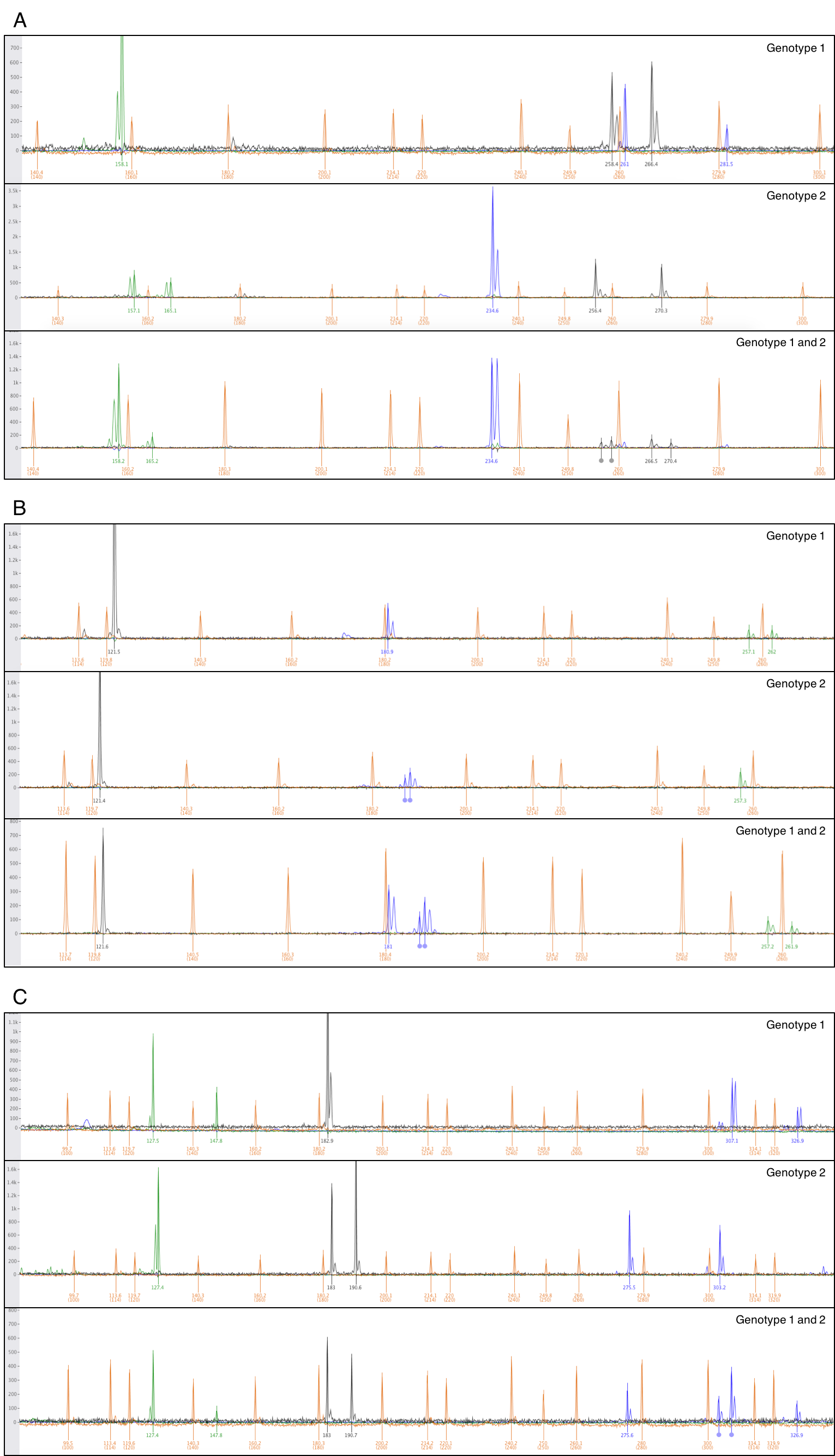

Supplement: File S3 — These peaks were output from Geneious after fragment analysis on an ABI 3130xl capillary DNA sequencer. A: Psp_02 (blue), Psp_23 (green), and Psp_33 (black). B: Psp_16 (blue), Psp_29 (green), and Psp_39 (black). C: Psp_18 (blue), Psp_32 (green), and Psp_48 (black). Specific information for each locus is shown in Table 1. [file peerj-06-5915-s003.png]
